# Supplementary material for: Implementation of a Provincial Long COVID Care Pathway in Alberta, Canada: Provider Perceptions
Source: Healthcare (Basel). 2024 Mar 27;12(7):730. doi: 10.3390/healthcare12070730 (PMC11011656; doi:10.3390/healthcare12070730)
Supplement: Supplementary file 1 [file healthcare-12-00730-s001.zip › Supplemental Table S1 Structured Guide v1-081023.pdf]

SUPPLEMENTAL TABLE S1

Thank you for taking the time to share about your current sites processes and readiness on implementing the post-COVID pathway and post-COVID functional scale. As noted in the informed consent form, your participation is entirely voluntary. You do not have to answer any question if you do not want to, and we can stop at any time.

Our study is part of a federally-funded, provincial evaluation of whether the incorporation of the Post-COVID Rehabilitation Framework (PCRF) can improve the screening and assessment of post-COVID recovery needs. Two major tools within the PCRF include a symptom specific screening tool (adopted from the C19-YRS) and assessment tool (Post COVID functional scale or PCFS).

This particular interview aims to learn more about the organizational supports and processes of early adopter sites that will support the evaluation of the PCRF implementation. We want to understand your site's implementation needs so we can tailor your site-specific implementation strategy and evaluation. The information from this interview will also help us compare site readiness before and after the implementation of the Post-COVID Rehabilitation Framework.

We expect that this interview will take about 20-30 minutes. If you would like to take a break for any reason during the interview, please let us know.

This interview will be recorded so that we can take notes of your answers, however, your name and any other personal information will remain confidential. Is this alright with you?

Do you have any questions or concerns before we begin?

I am first going to ask a few questions about your site or team:

1. What is the name of your site or team

2. What are the current number (or approximate number) of professionals associated with your site?

3. What types of professionals work at your site (e.g., physician, OT, PT, admin)?

4. What are the characteristics of the patient population that receive care from your site?

5. Where does your site fall on the care continuum?
    a. **Choices**: *Primary care, acute care, inpatient care, outpatient care*

6. How do patients come to your site?
    a. **Prompt:** *What is the referral process? How do patients find out about your clinic?*

7. How does your site currently assess patients with post-COVID conditions?
    a. **Prompt***: PCFS, partial PCFS, in combination with other assessment tools? Other?*

8. How would you classify/diagnose a post-COVID patient (i.e. # weeks following acute infection)?

9. What types of post-COVID patients does your site see?
    a. **Prompt:** *Mild functional impairment; Moderate functional impairment; Severe functional impairment*

10. How does your site follow up with patients with post-COVID needs? (*referrals, call backs*)

    a. **Prompt**: *How does your site support patient wayfinding to appropriate rehabilitation resources?*

I am now going to ask you questions about your perception of the PCRF, and PCRF implementation

11. How likely do you think it is that implementing a post-COVID pathway - or tools within the PCRF such as the PCFS - will lead to better outcomes for post-COVID-19 patients?
    Prompts
    a. What do you think are the benefits of doing this, for A. You? B. Your practice?
    b. In your opinion, do the benefits of implementing the PCRF outweigh the costs?

12. How confident are you that your team can access the screening, assessment, and supports for persons with Post COVID Conditions such as the pathways, tools, and resources within the PCRF? (e.g., PCFS)
    Prompts
    a. Are you currently using the PCFS or plan on using it in the future?

13. Are you aware of any conflicting beliefs about Post-COVID Conditions or the PCFS in your care context?

If yes to Q12- start here (Site IS using the PCRF or PCFS)

14. How does your site currently use the pathways, tools, and resources within the PCRF?
    a. **Prompt**: *The PCRF contains screening and assessment tools as well as self-management resources. Ask about clinical pathways and resources*

15. How does your site capture PCFS data?

16. Where is PCFS data stored?

17. What role administers the PCFS at your site?

18. How easy or difficult is it to remember the steps involved in the PCRF/PCFS in daily practice?

19. Have you received feedback about your site's use of the PCRF? From patients? From colleagues?

20. To what extent do you intend to continue to use the PCFS/PCRF tools in daily clinical practice?
    a. **Prompt**: *Are there things you need to do before you can do this?*

If 'NO' or 'planning to use' to Q12- start here (Site is NOT using the PCRF or PCFS)

21. To what extent do you intend to use the PCFS/PCRF tools in daily clinical practice?
    Prompts
    a. What is preventing your site from using the PCRF?
    b. What do you need in order to start using it?
    c. Are there things you need to do before you can do this?

22. How do you anticipate administering the PCFS at your site?
    Prompts:
    a. Which roles do you see as supporting the administration of the PCFS?
    b. *Are there things you need to do before you can do this?*
    c. *Does the current infrastructure, staff capacity, or other supports allow you to do this?*

23. How would you anticipate your site would store PCFS data?
    a. ***Prompt****: Are there things you need to do before you can do this?*

24. Have you heard any feedback from colleagues or other sites about use of the pathways, tools, and resources within the PCRF?

25. What timing of PCRF implementation and post-implementation data collection would be ideal for your site?
    What we mean by this is when do you want the 6-month period to data collection to be? Ex secondary data and

26. Do you have any questions or anything else you would like to add?

END INTERVIEW
