# Supplementary material for: Implementation of a Provincial Long COVID Care Pathway in Alberta, Canada: Provider Perceptions
Source: Healthcare (Basel). 2024 Mar 27;12(7):730. doi: 10.3390/healthcare12070730 (PMC11011656; doi:10.3390/healthcare12070730)
Supplement: Supplementary file 1 [file healthcare-12-00730-s001.zip › Supplemental Table S2 Semi-Structured Guide v1-080923.pdf]

SUPPLEMENTAL TABLE S2

PCRF EVALUATION: FOCUS GROUP SCRIPT AND QUESTION GUIDE

INTRODUCTION

Thank you all for agreeing to join us today. This interview is part of a broader ongoing evaluation of the implementation of a coordinated, provincial rehabilitation response framework to address the recovery from, and sequelae of, COVID-19. The framework was developed in Spring 2021 by a provincial task force, and was called the post-COVID rehabilitation framework (or 'The Framework').

Our aim for today is to discuss your experience of providing care to individuals recovering from COVID-19, including those with post-COVID conditions. We want to learn about your perceptions and use of the screening tools, pathways, and resources available for providing care to people with these conditions. Learning from you will help with the broader implementation of the post-COVID rehabilitation framework, which encompasses those tools, pathways and resources.

We will be recording today's meeting to help with documenting and analysis of today's discussion. Any identifying information that may be in the recording will be removed from the transcript. Is this ok with everyone?

[START RECORDING]

Your decision to participate today is voluntary. If you no longer wish to take part you may excuse yourself at any time, and if you would prefer not to answer a particular question, that is completely acceptable. Everyone participating today has signed a confidentiality agreement and we are all responsible for respecting and protecting each other's privacy.

QUESTIONS

1. Can everyone share what is their current role?
    a. What are your years of professional experience,
    b. Can you tell me a bit about your journey to get to where you are now?

2. In your practice, if you suspect your patient is presenting with post-COVID symptoms, what is your current approach to screening and assessing their symptoms?
    a. How would you generally describe the types of post-COVID patients you come across in your practice? (i.e., severity, types of symptoms, duration of symptoms)

3. In your practice, what do you see as the current challenges to screening and assessing post-COVID conditions?
    a. What could make this easier for you and your team?
    b. Do you feel patients with post-COVID have the ability to access health services?
    c. Would it be helpful for your own practice to know about how other clinics, sites, or practices in Alberta screen and assess for post-COVID conditions?
    d. Where is the healthcare system doing well? Where might the healthcare system improve?

Let's talk about tools for screening and assessment for post-COVID conditions:

4. We heard that you have recently received training on the post-COVID functional scale or PCFS.
    a. Have you started to use the PCFS?
    b. What do you like about it?
    c. What do you not like about it?
    d. Is there anything you would change?

[If NO to Q4a, proceed to question 5. If YES, skip to Q6]

5. What would you need to use the PCFS to support patients with post-COVID (for example, further training, support, resources?)?
    a. Is there anything that prevents you from fully utilizing these resources?

Let's talk about the available clinical pathways for post-COVID screening and assessment

The clinical pathway refers to the 'Adult Long COVID Pathway', which is a decision making tool for providers. The post-COVID pathway is centered on using the PCFS to determine a patient's level of functional impairment, which can help providers recommend the appropriate type and intensity of rehabilitation required.

6. Are you familiar with a post-COVID clinical pathway being used at your site?

7. Please describe your experience of using the post-COVID clinical pathway at your site to assess and post-COVID symptoms.
    a. How confident do you feel using the clinical pathway to triage patients?
    b. Are the steps of the pathway easy to follow? Why or why not?
    c. What is the advantage of using a clinical pathway to triage patients with post-COVID?
    d. What are the disadvantages?
    e. What would you change about the current post-COVID clinical pathways?

Let's talk about the available post-COVID resources for patients and providers

8. Are you familiar with the currently available patient-facing post-COVID educational materials or resources (e.g., websites, government resources, social media)?
    a. What resources have you or do you provide to patients and why?
    b. Do you have any thoughts on how existing educational materials or clinical for patients could be improved?
    c. How did you originally learn of or come across these resources?

9. Are you familiar with the currently available provider educational materials or resources (e.g., websites, informational sessions, meetings, workshops)?

a. What other types of resources would you like to see?
b. Were there any attempts at education that fell flat, or were not helpful?
c. Are there any areas you would like more education or support?
d. Do you have any thoughts on how existing educational materials or clinical pathways for providing care to patients could be improved?
e. How did you originally learn of or come across these resources?

10. Is there anything else you would like to add that we haven't touched on today?

Thank you for your time!
